# Controlling the Goos-Hänchen shift in a double prism structure using three-level Raman gain medium

**DOI:** 10.1038/s41598-023-50254-6

**Published:** 2023-12-20

**Authors:** Saeed Asiri, Li-Gang Wang

**Affiliations:** 1 Institute of Quantum Technologies and Advanced Computing, KACST, Riyadh, 11442 Saudi Arabia; 2 School of Physics, Zhejiang University, Hangzhou, 310058 China

**Keywords:** Quantum optics, Quantum physics

## Abstract

We propose a scheme to control the Goos-Hänchen (GH) shift of TE and TM reflected light beams in a double-prism structure, where a three-level Raman gain medium is filling the gap between the two prisms. We find that it is possible to control the GH shift in this structure by externally adjusting the optical properties of the Raman gain atomic medium while the gap width between the two prisms is fixed. Inspired by recent successful implementation of the double-prism configuration with an air gap to measure the GH shift, we expected that our proposal to control the GH shift can be achieved experimentally and used in different potential applications of the GH shift.

## Introduction

Geometrical optics predicts that at the boundary between two different media, an incident light beam reflects back to the first medium exactly from the same point of incidence. In fact, an incident light beam from a medium with refractive index $n_{1}$ suffers a lateral shift in comparison to the geometrical optics prediction when totally internally reflected from a medium with a refractive index $n_{2} < n_{1}$. This lateral shift which occurs at the interface between the two media is called Goos-Hänchen (GH) shift^1–9^.

Lateral GH shift has been a significant research topic in the recent years due to its possible applications in different fields. This phenomena has been shown to occur in various configurations and materials such as photonics crystals^10–12^, dielectric slabs^6,13^, and metamaterials^14–16^. It has also been investigated at the interface of Weyl semimetals^17,18^ and in structures containing graphene^19–24^. In a recent study, giant GH shift has been demonstrated to occur at the interface of PT-symmetric bilayer structure due to surface modes^25^. On the other hand, atomic coherence effects which occur in many atomic systems have been widely employed to coherently control the GH shift^26–36^. Because the dispersion as well as the absorption in such systems can be controlled, different proposals based on this idea have been considered to control the GH shift. The first proposal to control the GH shift using an atomic medium was considered using a Fabry-Perot cavity filled by a two- level atomic medium^26^, it was shown that the GH shift can be positive or negative by only changing the driving field intensity. Following this work^26^, other different atomic systems have been implemented and showed that the lateral GH shift can be externally controlled using different parameters and properties in each of these these systems^26–36^.

GH shift has been considered previously in structures involving one prism^37–39^ and two prisms^40–46^ configurations. In a system containing two prisms separated by an air gap^40^, GH shift of a reflected microwave has been experimentally measured and shown to be affected by the beam width and the angle of incidence. More recently, the effect of spatial coherence and beam width on the GH shift for a partially coherent light beam in a double-prism system was experimentally studied^47^. GH shift for a reflected beam in a similar arrangement has been analyzed using the stationary phase theory^9^ and energy flux method^42^. Various materials have been considered to fill in the gap in the double-prism structure such as negativezero-positive index metamaterial^43^.

In this paper, we consider the coherent control of the GH shift in a double-prism structure where a three-level atomic medium is placed between the two prisms. Having air or other fixed refractive index materials between the prisms results in lateral shifts that can be modified by only changing the angle of incidence or the gap width between the two prisms. We show that even if the gap width between the two prisms as well as the angle of incidence are fixed, the GH shift can still be controlled by filling the gap between the two prisms by a coherently controlled refractive index medium. Such quantum-optics method enables us to flexibly control the optical GH shift in a fixed configuration and in turn probe precisely the change in light-matter interactions.

## Model

**Figure 1:**
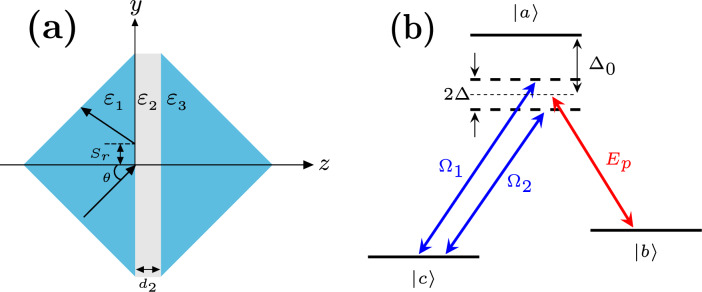
(**a**) Schematic of a double prism structure separated by a gap of width $d_{2}$, where an atomic medium is filling the gap. (**b**) Energy levels diagram of the Raman gain atomic medium.

We consider the GH shift of a probe beam reflected from a double prism structure as shown in Fig. 1a. We assume that the probe beam is incident from the left prism whose permittivity $\varepsilon _{1}$ is equal to the permittivity of the second prism $\varepsilon _{3}$. The two prisms are separated by a gap of width $d_{2}$, and filled by a Raman atomic gain medium with permittivity $\varepsilon _{2}$. The permittivity of the atomic medium $\varepsilon _{2}$ can be controlled by some external parameters of the medium. Therefore, the GH shift in this system can be controlled via modifying the optical properties of the atomic medium.

The GH shift of the reflected beam can be calculated using the stationary phase theory^9^ as

1 $$\begin{aligned} S^{p}_{r} = - \dfrac{1}{k \sqrt{\varepsilon _{1}} \cos \theta } \frac{d \phi ^{p}_{r}}{d \theta }, \end{aligned}$$

where *k* is the wave number in free space and the superscript $p = \textrm{TE, TM}$ represents the two linear polarization states of the incident beam, while the subscript *r* indicates that Eq. (1) is used to calculate the GH shift for the reflected beam. $\phi ^{p}_{r} = atan\Big (\textrm{Im}(r^{p})/\textrm{Re}(r^{p}) \Big )$ is the phase shift associated with the reflected beam for the two different polarizations, and $r^{p}$ is the reflection coefficient of the reflected beam.

The reflection coefficient of the p-polarized beam is given by

2 $$\begin{aligned} r^{p}(\theta , \omega ) = \dfrac{r^{p}_{12} + r^{p}_{23} \; e^{2i k_{z2} d_{2}}}{1 + r^{p}_{12} r^{p}_{23} e^{2i k_{z2} d_{2}}}, \end{aligned}$$

where the Fresnels’ coefficients $r^{p}_{ij}$ at each interface for the TE and TM polarization cases are given by

3a $$\begin{aligned} r^{TE}_{ij}&= \dfrac{k_{zi} - k_{zj}}{k_{zi} + k_{zj}}, \end{aligned}$$

3b $$\begin{aligned} r^{TM}_{ij}&= \dfrac{\dfrac{k_{zi}}{\varepsilon _{i}} - \dfrac{k_{zj}}{\varepsilon _{j}}}{\dfrac{k_{zi}}{\varepsilon _{i}} + \dfrac{k_{zj}}{\varepsilon _{j}}}, \end{aligned}$$

where $i = 1, 2$ and $j = 2, 3$. In Eqs. (3a) and (3b), $k_{z1} = k_{z3} = k \sqrt{\varepsilon _{1}} \cos \theta$ is the wave vector in the first and second prisms along the z-direction, while $k_{z2} = k \sqrt{\varepsilon _{2} - \varepsilon _{1} \sin ^2 \theta }$ is the wave vector in the gap between the two prisms.

## Raman gain medium

Raman gain medium was experimentally realized^48^ using a gas of caesium atoms to show that the group velocity of a light pulse propagating in this medium can be larger than the speed of light in vacuum due to the negative dispersion of the light pulse in this gas medium. It has also been employed using a rubidium gas medium to demonstrate wide bandwidth white light cavity^49^. Raman medium is a $\Lambda$-type atomic system with an excited state $\vert a\rangle$ and two ground states $\vert b\rangle$ and $\vert c\rangle$ as shown in Fig. 1b. Two driving fields with Rabi frequencies $\Omega _{1}$ and $\Omega _{2}$ are set to couple the $\vert a\rangle \leftrightarrow$ $\vert c\rangle$ transition, where both fields are far detuned from the transition frequency $\omega _{ac}$ of the $\vert a\rangle \leftrightarrow$ $\vert c\rangle$ transition. The driving fields $\Omega _{1}$ and $\Omega _{2}$ are detuned from the $\vert a\rangle \leftrightarrow$ $\vert c\rangle$ transition by $\Delta _{0} - \Delta$ and $\Delta _{0} + \Delta$, respectively. The detunings are defined such that $\Delta _{0} = \omega _{ac} - (\nu _{1} + \nu _{2})/2$ and $\Delta = (\nu _{1} - \nu _{2})/2$, where $\nu _{1}$ and $\nu _{2}$ correspond to the frequencies of the first and second driving fields. A probe field with frequency $\nu _{p}$ and Rabi frequency $\Omega _{p}$ couples the $\vert a\rangle \leftrightarrow$ $\vert b\rangle$ transition. By assuming weak probe of the medium, the polarization of the medium can be calculated to the lowest order of the probe field and the susceptibility of the atomic medium $\chi$ can then be obtained^50^. The permittivity of the atomic medium is defined in terms of its dielectric susceptibility as $\varepsilon _{2} = 1 + \chi$, where $\chi$ is given by

4 $$\begin{aligned} \chi = \dfrac{M_{1}}{(\delta _{p} - \Delta ) + i \Gamma } + \dfrac{M_{2}}{(\delta _{p} + \Delta ) + i \Gamma }, \end{aligned}$$

where $M_{1, 2} = A \dfrac{|\Omega _{1, 2}|^2}{\Delta _{0}^{2}}$, $\Delta _{0} = (\Delta _{1} + \Delta _{p})/2$, $\Delta _{1} = 2 \pi (\nu _{1} - \nu _{ca})$, $\Delta _{p} = 2 \pi (\nu _{p} - \nu _{0})$, $\delta _{p} = \nu _{p} - \nu _{0}$, and $\nu _{0} = \nu _{1} - \nu _{ca} + \nu _{cb}$. *A* is the number density of atoms and $\Gamma$ represents the spectral width of two gain lines. For simplicity, we consider that $\Omega _{1} = \Omega _{2}$ which consequently gives $M_{1} = M_{2}$. It is clear from Eq. (4) that the susceptibility of the Raman gain medium and hence its refractive index can be modified and controlled by changing several parameters such as the Rabi frequencies of the driving fields $\Omega _{1}$ and $\Omega _{2}$ and the probe field detuning $\delta _{p}$. The incident probe field when resonantly interacting with the Raman gain medium experiences zero dispersion and small amount of gain. For all the selected values of the Rabi frequencies of the driving fields $\Omega _{1}$ and $\Omega _{2}$ in this work, the probe field still has no dispersion as long as it is resonantly interacting with the medium. On the other hand, the gain in the probe field at resonance $\delta _{p} = 0$ becomes larger when the Rabi frequencies of the driving fields increase. Slightly away from resonance and between the two Raman gain doublet, the probe field dispersion becomes negative. The optical properties of the Raman gain medium can obviously be controlled and this is the motivation of using this system to coherently control the GH shift in this work.

**Figure 2:**
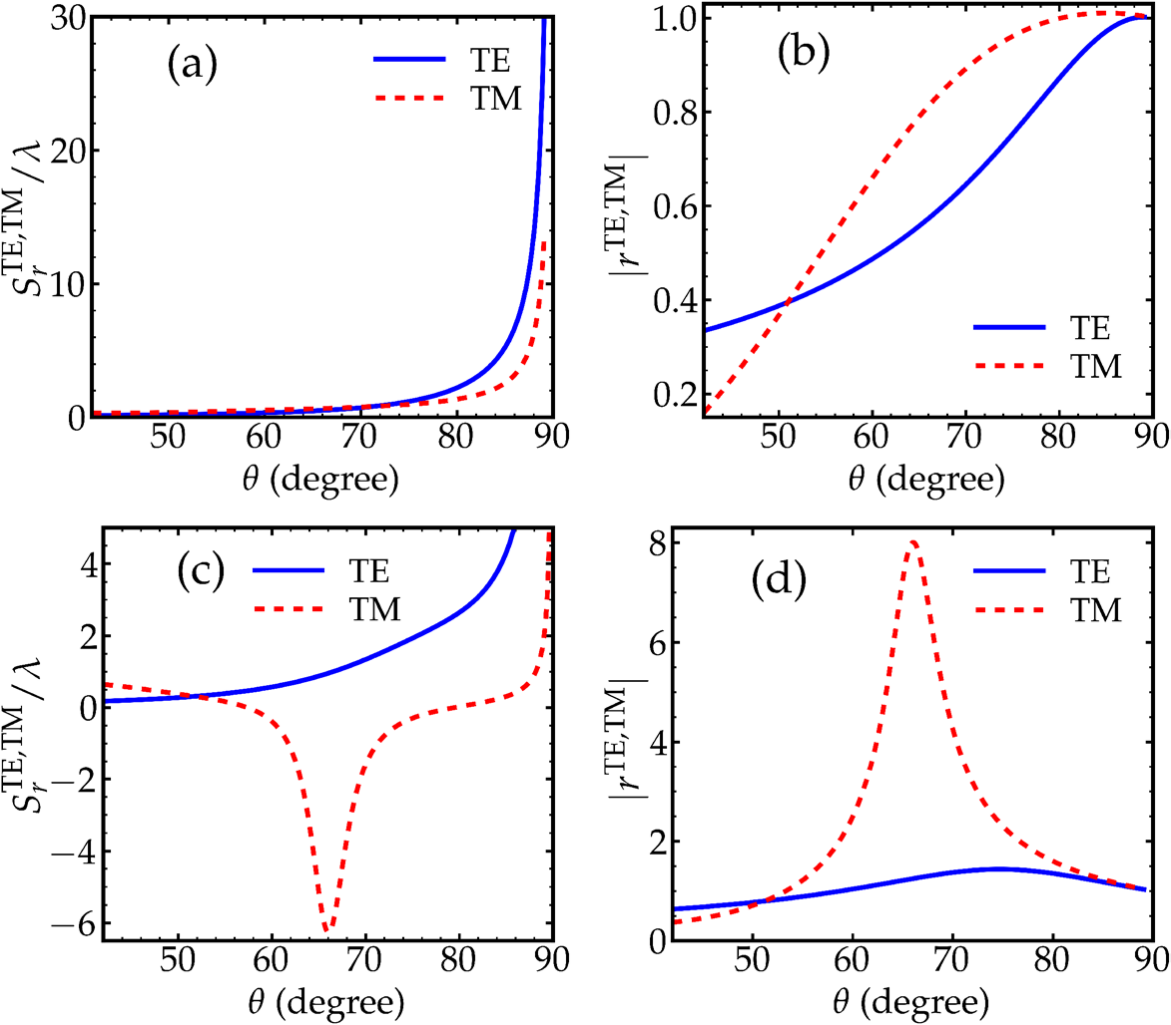
(**a**) and (**c**) GH shifts of the reflected TE and TM fields versus the angle of incidence. (**b**) and (**d**) absolute value of the reflection coefficients of the TE and TM fields versus the angle of incidence. In (**a**) and (**b**), $\Omega _{1} = \Omega _{2} = 2 \gamma$. In (**c**) and (**d**), $\Omega _{1} = \Omega _{2} = 8 \gamma$. The probe field detuning is $\delta _{p} = 0$. Other parameters are $\gamma = 1 \textrm{MHz}$, $A =\gamma$, $\Gamma = 0.8 \gamma$, $\Delta _{1} = 5 \gamma$, $\Delta _{p} = 4.9 \gamma$, $\Delta _{\nu } = 1.8 \gamma$, $\omega _{p}/2\pi = 300 \textrm{THz}$, and $d_{2} = 0.1 {\mu }\textrm{m}$.

## Results

Based on the fact that the optical properties of the Raman medium can be controlled, we predict that the GH shift of the reflected probe beam can be modified and controlled. In Fig. 2, the GH shifts and the corresponding reflectivities for the TE-polarized as well as the TM-polarized probe fields are shown as a function of the angle of incidence. The gap width between the two prisms is fixed i. e., $d_{2} = 0.1 {\mu }\textrm{m}$. We also assume that the probe field detuning $\delta _{p} = 0$, where in this case the Raman medium exhibits no dispersion to the probe field except minimal amount of gain. As shown in Fig. 2a, the GH shifts for the two polarization cases are nearly identical when the Rabi frequencies of the driving fields are $\Omega _{1} = \Omega _{2} = 2 \gamma$. Fig. 2b shows the reflectivity for these values of the driving fields frequencies where it exhibits smooth behavior. On the other hand, when these frequencies are increased to $\Omega _{1} = \Omega _{2} = 8 \gamma$ as shown in Fig. 2c, the GH shift for the TE-polarized field preserves the same behavior as in Fig. 2a while for the TM-polarized field, the GH shift becomes negative nearly between the angles $60^{\circ }$ and $70^{\circ }$. The negative dip which appears in the GH shift for this case is due to a sudden change in the phase $\phi ^{TM}_{r}$ of the reflected TM beam. Fig. 2d, shows a large peak in the reflectivity of the TM case where the negative GH shift is exactly located as shown in Fig. 2c.

### Effect of gap width on GH shift

**Figure 3:**
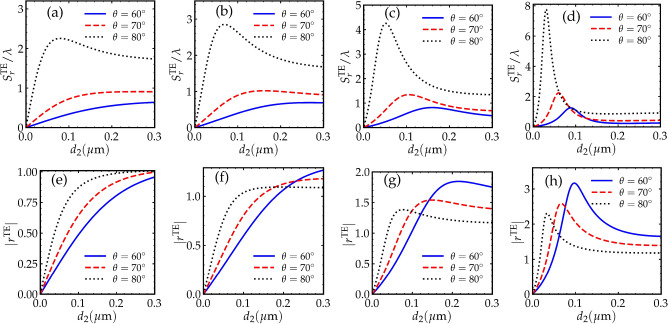
(**a**)–(**d**), GH shifts for the TE-polarized reflected beams as a function of the gap width $d_{2}$. (**e**)–(**h**) correspond to the absolute value of the reflection coefficients of the TE-polarized beams. In (**a**) and (**e**), $\Omega _{1} = \Omega _{2} = 2 \gamma$. In (**b**) and (**f**), $\Omega _{1} = \Omega _{2} = 5 \gamma$. In (**c**) and (**g**), $\Omega _{1} = \Omega _{2} = 8 \gamma$. In (**d**) and (**h**), $\Omega _{1} = \Omega _{2} = 12 \gamma$. Other parameters are the same as in Fig. 2.

**Figure 4:**
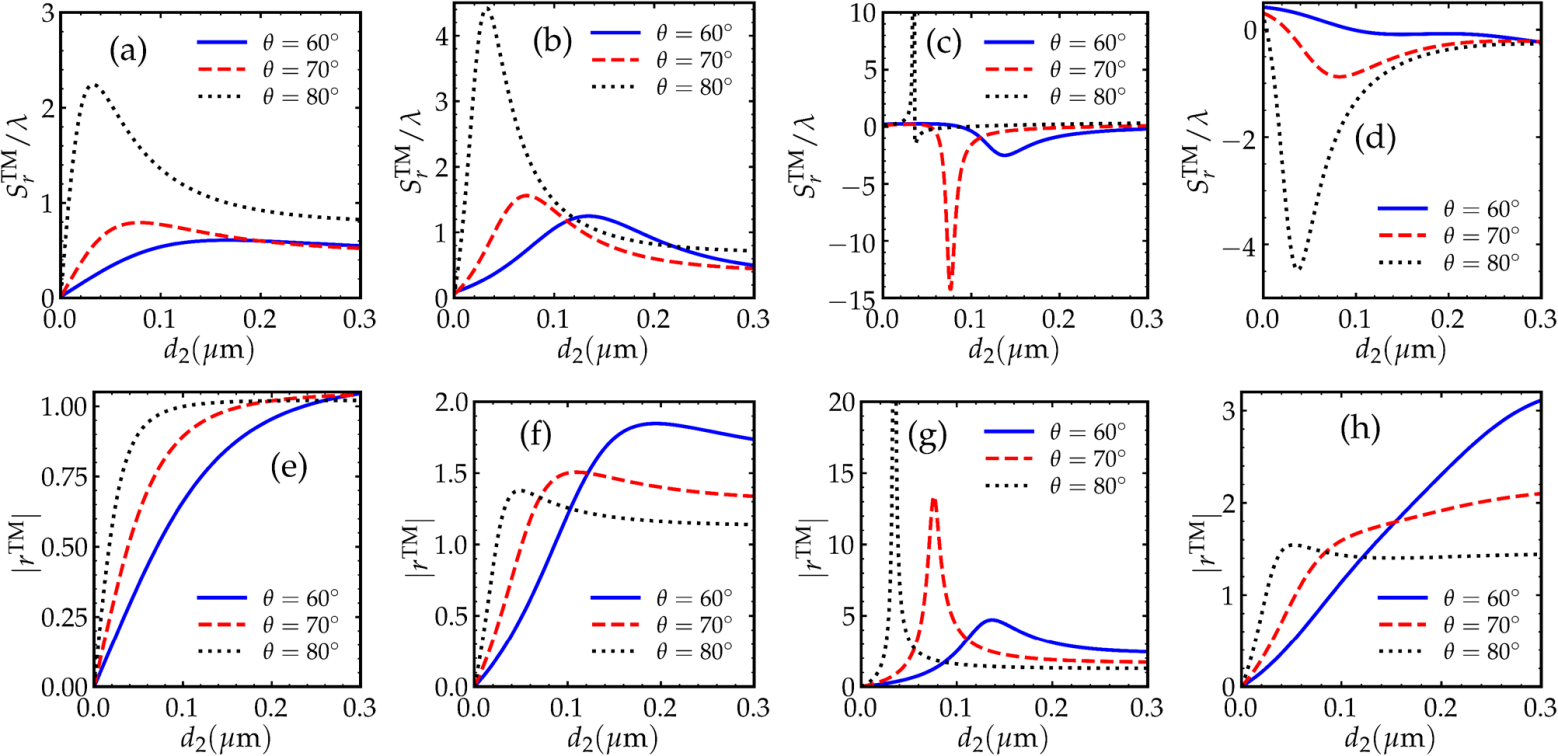
(**a**)–(**d**) show the GH shifts for the TM-polarized reflected beams as a function of the gap width $d_{2}$. (**e**)–(**h**) show the absolute value of the reflection coefficients for the TM-polarized reflected beams as a function of the gap width $d_{2}$. In (**a**) and (**e**), $\Omega _{1} = \Omega _{2} = 2 \gamma$. In (**b**) and (**f**), $\Omega _{1} = \Omega _{2} = 5 \gamma$. In (**c**) and (**g**), $\Omega _{1} = \Omega _{2} = 8 \gamma$. In (**d**) and (**h**), $\Omega _{1} = \Omega _{2} = 12 \gamma$. Other parameters are the same as in Fig. 2.

The gap width between the two prisms plays an important role in determining the amplitude and even the direction of the GH shift. In Fig. 3, the GH shift as well as the reflectivity of the reflected TE-polarized field are plotted as a function of the gap width $d_{2}$ for three angles of incidence. We choose four different values of the Rabi frequencies of the driving fields in order to examine their effects on the GH shift while the probe field is resonantly interacting with the Raman medium. In Fig. 3a,b,c,d, the amplitude of the GH shift obviously increases as the angle of incidence is increased. It can also be noticed that the GH shift increases as the driving fields intensities are increased for the same angle of incidence, and that is due to the increasing gain at resonance in the Raman medium for larger driving intensities. The absolute value of the reflection coefficients for all the selected values of $\Omega _{1}$ and $\Omega _{2}$ are plotted in the second row of Fig. 3. By examining these reflectivity curves, one can intuitively expect the values of the gap width where the GH shift can be maximum or when it reaches constant value. In the double-prism configuration, total internal reflection can be frustrated^40,44^ by bringing the second prism extremely close the the first prism, where in this case the evanescent energy starts to propagate through the second prism. In Fig. 3a,b,c,d, it is interesting to see that for significantly small gap width in comparison to the incident wavelength, the GH shift can be below an order of a wavelength due to the frustrated total internal reflection. When the second prism becomes slightly away from the first one, we observe rapid change in the GH shift in which it reaches a maximum value that can be magnified by increasing the driving fields intensities. When the gap width becomes large i.e., $d_2 > 0.1 \mu \textrm{m}$, the second prism can no longer affect the GH shift.

Figure 4 shows the GH shift for the reflected TM-polarized field along with the reflectivity of the field. The GH shifts when the driving field intensities $\Omega _{1} = \Omega _{2} = 2 \gamma$ and $\Omega _{1} = \Omega _{2} = 5 \gamma$ are shown in Fig. 4a,b, respectively. The GH shift in this case is positive over all the selected range of $d_2$ with slightly larger amplitude than amplitude of the TE polarization case for the same driving fields intensities. At the angle of incidence $\theta = 80^\circ$ in Fig. 4a,b, the GH shift rapidly increases and reaches its maximum value within a small gap width in which the two prisms are very close to each other i.e., $d_{2} < 0.1 \mu \textrm{m}$ in comparison to the case of TE-polarized field in Fig. 3a,b. Therefore, frustrated total internal reflection in the case of TM-polarized field requires smaller distance between the two prisms than in the case of TE-polarized field. When $\Omega _{1} = \Omega _{2} = 8 \gamma$ and $\Omega _{1} = \Omega _{2} = 12 \gamma$, the GH shift for the TM polarization can be switched to negative under the effect of the driving fields as shown in Fig. 4c,d. These large negative shifts are attributed to different sharp phase shifts that occur around the same values of $d_2$ where large negative GH shifts are observed. Therefore, observing negative GH shift in this system requires an incident TM-polarized probe beam and Rabi frequencies of the driving fields $\Omega _{1} = \Omega _{2} = 8 \gamma$ or larger. Interesting to see that changing the response of the atomic medium using the driving fields results in switching the direction of the GH shift for this polarization case. The reflectivities corresponding to the GH shifts in Fig. 4a,b,c,d are plotted in Fig. 4e,f,g,h, respectively. It can obviously be noticed from these figures that the sharp changes in the reflectiveties are occurring at the same values of the gap width where the GH shift is magnified. Thus, under suitable gain conditions (i.e., suitable values of $\Omega _{1} = \Omega _{2}$), there exists resonant coupling between the atomic medium and evanescent waves in the double prism system, which leads to the enhancement of the GH shifts in the TM-polarized cases.

### Effect of probe field detuning on GH shift

**Figure 5:**
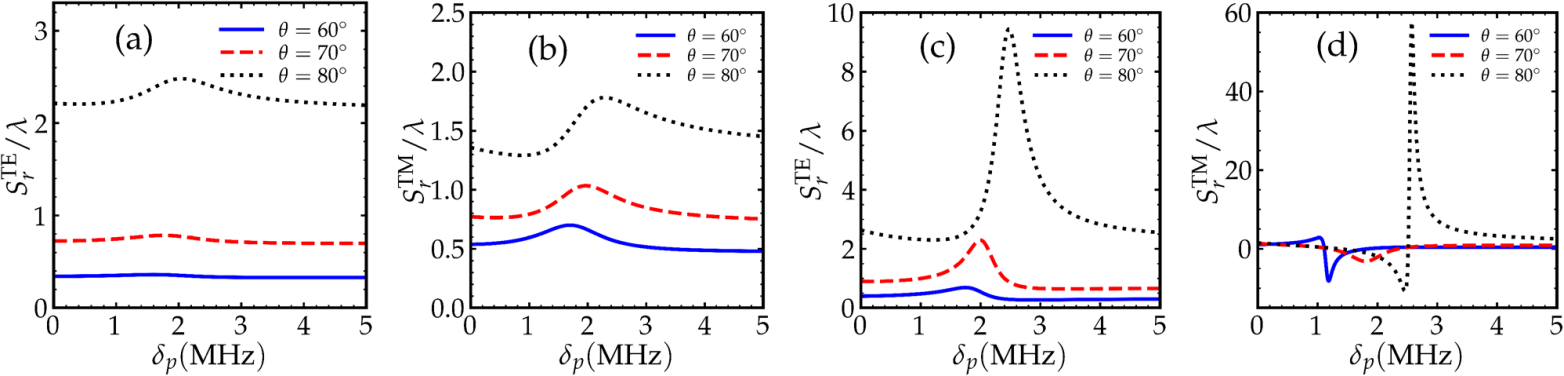
GH shift as a function of the probe field detuning for the TE (**a**, **b**) and TM (**c**, **d**) polarizations. In (**a**) and (**c**), $\Omega _{1} = \Omega _{2} = 2 \gamma$. In (**b**) and (**d**), $\Omega _{1} = \Omega _{2} = 5 \gamma$. Other parameters are the same as in Fig. 2.

The probe field detuning $\delta _{p}$ as mentioned previously is an important controlling parameter in the Raman gain medium, where changing $\delta _{p}$ modifies the dispersion as well as the gain in the probe field. In the previous parts, we focused on the resonant case $\delta _{p} = 0$, in which there is no dispersion. Here we show that changing the probe field detuning leads to modified amplitude and even direction of the GH shift. Raman gain medium exhibits negative dispersion between the gain doublet which are located at $\delta _{p} \approx \pm 1.8$ MHz as mentioned in^29,33^. Around these values of the detuning, the dispersion of the probe field becomes positive and the gain in the probe field reaches its maximum values. We plot the GH shift versus the probe field detuning where we cover the regions of negative and positive dispersions to show that controlling the detuning of the probe field results in controllable GH shift. Fig. 5a,c show the GH shifts of the reflected TE-polarized fields as a function of the probe field detuning. In Fig. 5a, the Rabi frequencies of the driving fields are $\Omega _{1} = \Omega _{2} = 2 \gamma$. GH shift in this case is not significantly affected by the variation of of the detuning of the probe field. We only observe larger amplitude of the GH shift for larger angles of incidence. In Fig. 5c, we consider $\Omega _{1} = \Omega _{2} = 5 \gamma$ where in this situation, the GH shift is significantly changing when the probe field detuning is modified. Fig. 5b,d show the GH shifts of the reflected TM-polarized fields as a function of the probe field detuning $\delta _{p}$. In Fig. 5b,d, the values of the Rabi frequencies of the driving field are similar to that in Fig. 5a,c, respectively. In comparison to the case of TE-polarized field, the GH shift for the case of TM-polarized field is greatly affected by changing the detuning of the probe field. GH shift is larger for the TM case and can also be negative as seen in Fig. 5d.

## Conclusion

In conclusion, we investigated the coherent control of the GH shift for a reflected light beam in a double-prism structure where the space between the prisms is occupied by a coherently controlled three-level Raman gain atomic medium. The motivation of selecting the double-prism arrangement to measure the GH shift of the reflected beam is the feasible experimental implementation of this system. Our results indicate that achieving coherent control of the GH shift is promising using the Raman gain medium. It is also expected that our proposal can be implemented experimentally especially when considering previous successful experiments on the GH shift using the two prisms system separated by air gap. The results here will provide the application of the GH shift in probing the atomic properties and controlling optical shifts in fixed configurations.

## Data Availability

The datasets that support the plots in this paper are available from the corresponding author on reasonable request.
